# Sevoflurane Exposure Exacerbates Memory Impairment and Pathological Manifestation by Inhibiting AKT/mTOR‐Mediated Autophagy in P301L Tau Transgenic Mice

**DOI:** 10.1002/cns.70694

**Published:** 2025-12-17

**Authors:** Kaiwu He, Zhijing Zhang, Youzhi Li, Wei Xiong, Yanmei Xing, Wenli Gao, Wei Kong, Lixin Chen, Xifei Yang, Zhongliang Dai

**Affiliations:** ^1^ Guangdong Provincial Clinical Research Center for Geriatrics, Shenzhen Clinical Research Center for Geriatrics, Department of Anesthesiology Shenzhen People's Hospital (The Second Clinical Medical College, Jinan University, The First Affiliated Hospital, Southern University of Science and Technology) Shenzhen China; ^2^ Guangdong Provincial Key Laboratory of Medical Immunology and Molecular Diagnostics Guangdong Medical University Dongguan China; ^3^ Shenzhen Key Laboratory of Modern Toxicology, Shenzhen Medical Key Discipline of Health Toxicology (2020–2024) Shenzhen Center for Disease Control and Prevention Shenzhen China; ^4^ Department of Anesthesiology, Affiliated Dongguan Hospital Southern Medical University (Dongguan People's Hospital) Dongguan China; ^5^ Department of Pharmacology, Medical College Jinan University Guangzhou China

**Keywords:** Alzheimer's disease (AD), autophagy, cognitive impairment, sevoflurane exposure, tau pathology

## Abstract

**Background:**

Alzheimer's disease (AD) is a global health event with progressive cognitive decline affecting millions of elderly people worldwide. Emerging evidence indicates that sevoflurane may be associated with AD progression. However, the underlying mechanism remains poorly understood. Herein, we explored the potential role of sevoflurane exposure in cognitive ability and the possible mechanisms of action in the early stage of Tg4510 (P301L tau) transgenic mice.

**Methods:**

Using the novel object recognition test and Y‐maze test, we assessed the change in cognitive abilities in 3‐month‐old male WT mice and Tg4510 mice with or without 3% sevoflurane exposure. By histological analysis and western blot, we determined the effect of sevoflurane exposure on tau pathology, synaptic function, and neuroinflammatory response. TMT‐labeled proteomics technique coupled with bioinformatics analysis was performed to identify the potential key pathways or proteins involved in sevoflurane‐induced cognitive deterioration.

**Results:**

Behavioral test showed that sevoflurane exposure exacerbated cognitive impairment of young Tg4510 mice. Pathologically, immunofluorescence demonstrated that sevoflurane exposure increased tau phosphorylation, synaptic defects, and neuroinflammatory response, which were further supported by the result of immunoblotting in Tg4510 mice exposed to sevoflurane. Proteomic analysis revealed an obvious decrease in autophagy‐related proteins including Sort1, Vps28, and Atg3 in the hippocampus of sevoflurane‐exposed Tg4510 mice compared to the nonexposed group. As an upstream signaling of autophagy, the AKT/mTOR pathway was found to be inhibited in sevoflurane‐exposed Tg4510 mice. Moreover, our data also validated an inhibition of autophagy signaling associated with sevoflurane exposure in the context of tau pathology, as indicated by the upregulated expression of p62 and the downregulated expression of Sort1, Vps28, and Atg3.

**Conclusion:**

These findings suggest that autophagy signaling appears to be a promising target for intervention in sevoflurane‐induced cognitive impairment in the early tau pathology, which lays the foundation for further study of the underlying mechanisms.

## Graphical abstract

Schematic diagram illustrating the exacerbating effect of sevoflurane exposure on memory impairment in the context of Tau pathology. Sevoflurane exposure increased tau phosphorylation, synaptic defects, and neuroinflammatory response of Tg4510 mice. Further, we found that sevoflurane‐induced cognitive impairment was linked with the inhibition of the AKT/mTOR‐mediated autophagy pathway, suggesting that interventions for this pathway might be a potential target for developing therapies to combat sevoflurane‐induced cognitive decline associated with tau pathology.

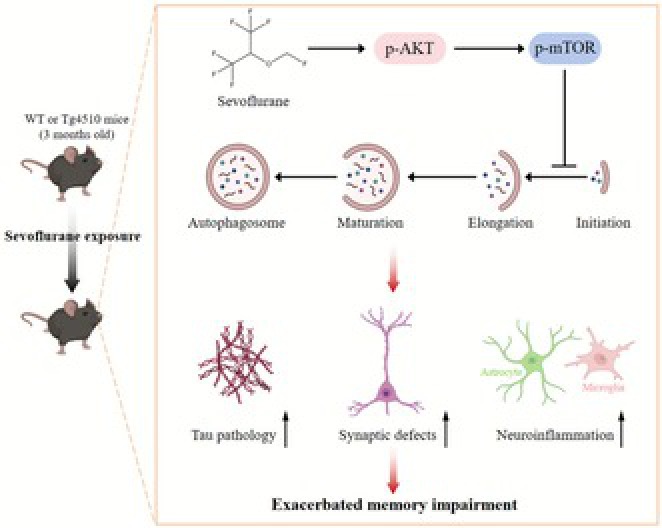

## 1 Introduction

Alzheimer's disease (AD) is the most common neurocognitive disorder accompanied by progressive memory decline, which greatly affects the health of millions of older adults worldwide. Despite extensive research efforts, no effective treatment or preventative strategies for AD have yet been identified. Thus, focusing on fundamental research on AD‐related cognitive impairment remains a top priority. Previous studies reveal that the combination of multiple AD‐related risk factors (including aging, *ApoE4*, *APP*, *PS1*, tau hyperphosphorylation, etc.) contributes to the onset and development of memory decline [1, 2, 3]. Sevoflurane, an inhalational anesthetic widely used in clinical practice, has recently been reported to have a certain degree of neurotoxicity [4, 5]. In animal experiments, sevoflurane was demonstrated to induce sequential tau phosphorylation, leading to cognitive impairment in young mice [6]. Meanwhile, sevoflurane could also impair synaptic plasticity and cognitive function in aged mice [7]. The above evidence indicates that sevoflurane may be a non‐negligible risk factor for cognitive impairment. Although the relationship between sevoflurane exposure and cognitive impairment is still controversial in the clinic, our previous studies found that clinical doses of sevoflurane exposure accelerated the onset of cognitive impairment and aggravated the neuroinflammatory response in Aβ‐driven AD transgenic mice [8, 9]. Consistently, similar results in the above model mice were also confirmed by multiple research groups [10, 11]. Several population‐based studies have found that sevoflurane can increase phosphorylated tau levels in the cerebrospinal fluid, thereby accelerating the progression of cognitive impairment [12, 13]. Therefore, it is necessary to explore the effect of sevoflurane exposure on cognitive impairment within the context of tau pathology.

Autophagy is an important cellular process that contributes to host cells eliminating misfolded, aggregated proteins and injured organelles, thereby promoting homeostasis, differentiation, development, and survival of neurons [14, 15]. Defects in autophagy are closely associated with the occurrence and development of multiple neurodegenerative diseases such as AD, Parkinson's disease (PD), and Amyotrophic lateral sclerosis (ALS) [16, 17, 18]. Thus, it is concluded that the enhancement of autophagy is an efficient strategy for improving AD‐related cognitive decline. Previous studies have uncovered that the elevated autophagy caused by rapamycin treatment can ameliorate cognitive deficits in sevoflurane‐exposed aged mice via improving autophagic flux [19]. In addition, autophagy activation significantly prevents sevoflurane‐induced neurotoxicity and increases cell viability in H4 human neuroglioma cells [20]. The above evidence indicates that the regulation of autophagy plays a key role in sevoflurane‐induced cytotoxicity. Besides, autophagy deficits are also observed in sevoflurane‐induced neuron apoptosis, Aβ pathology, and spatial learning impairment in APP/PS1 mice [21, 22]. However, the role of sevoflurane in autophagy associated with tau pathology remains unclear.

The AKT/mTOR signaling pathway plays a key regulatory role in autophagy [23]. In the initiation of autophagy, mTOR (an important target molecule sensing growth factor and nutrient signals) is closely associated with the onset of autophagy. For instance, the high expression of mTOR phosphorylation at serine 2448 under nutrient‐sufficiency conditions disrupts the interaction between Ulk1 and AMPK by reducing Ulk1 phosphorylation, thereby inducing the inhibition of autophagy [24]. As an upstream signal for mTOR, AKT is involved in the regulation of a variety of biological processes such as cell growth, proliferation, survival, differentiation, and metabolism [25]. In AD, a body of increasing evidence indicates that the inhibition of the AKT/mTOR signaling pathway may contribute to alleviating AD‐related cognitive impairment and pathological manifestations [26, 27]. Thus, we believe that AKT/mTOR‐mediated autophagy may play a pivotal role in the pathogenesis of AD. Previous studies show that the neurotoxicity caused by sevoflurane is at least partially mediated by the activation of the AKT/mTOR pathway in the brain of developing mice [28, 29]. Besides, an in vitro study demonstrates that autophagy activation can prevent sevoflurane‐induced neurotoxicity [20]. From this, we assume that sevoflurane‐induced neurotoxicity may be closely related to the inhibition of autophagy mediated by the AKT/mTOR pathway. However, whether the AKT/mTOR‐mediated autophagy associated with sevoflurane exposure is involved in the regulation of AD‐related cognitive impairment remains unclear.

In this study, we determined the deteriorative effect of sevoflurane exposure on cognitive function, tau phosphorylation, synaptic damage, and neuroinflammatory response in P301L tau transgenic mice. Further, our findings also elucidated a significant contribution of autophagy inhibition caused by activating the AKT/mTOR pathway in sevoflurane‐exacerbating cognitive impairment associated with tau pathology.

## 2 Materials and Methods

### 2.1 Animals

Tg4510 mice (129S6.Cg‐Tg(Camk2a‐tTA)1Mmay/JlwsJ; Fgf14Tg(tetO‐MAPT*P301L)4510Kha/J) and the wild‐type (WT) (129S6.Cg‐Tg(Camk2a‐tTA)1Mmay/JlwsJ) were obtained from Prof. Xifei Yang (Shenzhen Center for Disease Control and Prevention). The animal experiments were approved by the Animal Care and Use Committee of Shenzhen People's Hospital (approval number: AUP‐220516‐DZL‐0230‐01). Both male Tg4510 mice and WT mice were kept in a controlled environment with constant temperature and humidity and with food and water ad libitum. All experiments adhered to ethical guidelines for animal welfare and strived to reduce suffering as much as possible.

### 2.2 Sevoflurane Exposure

**FIGURE 1:**
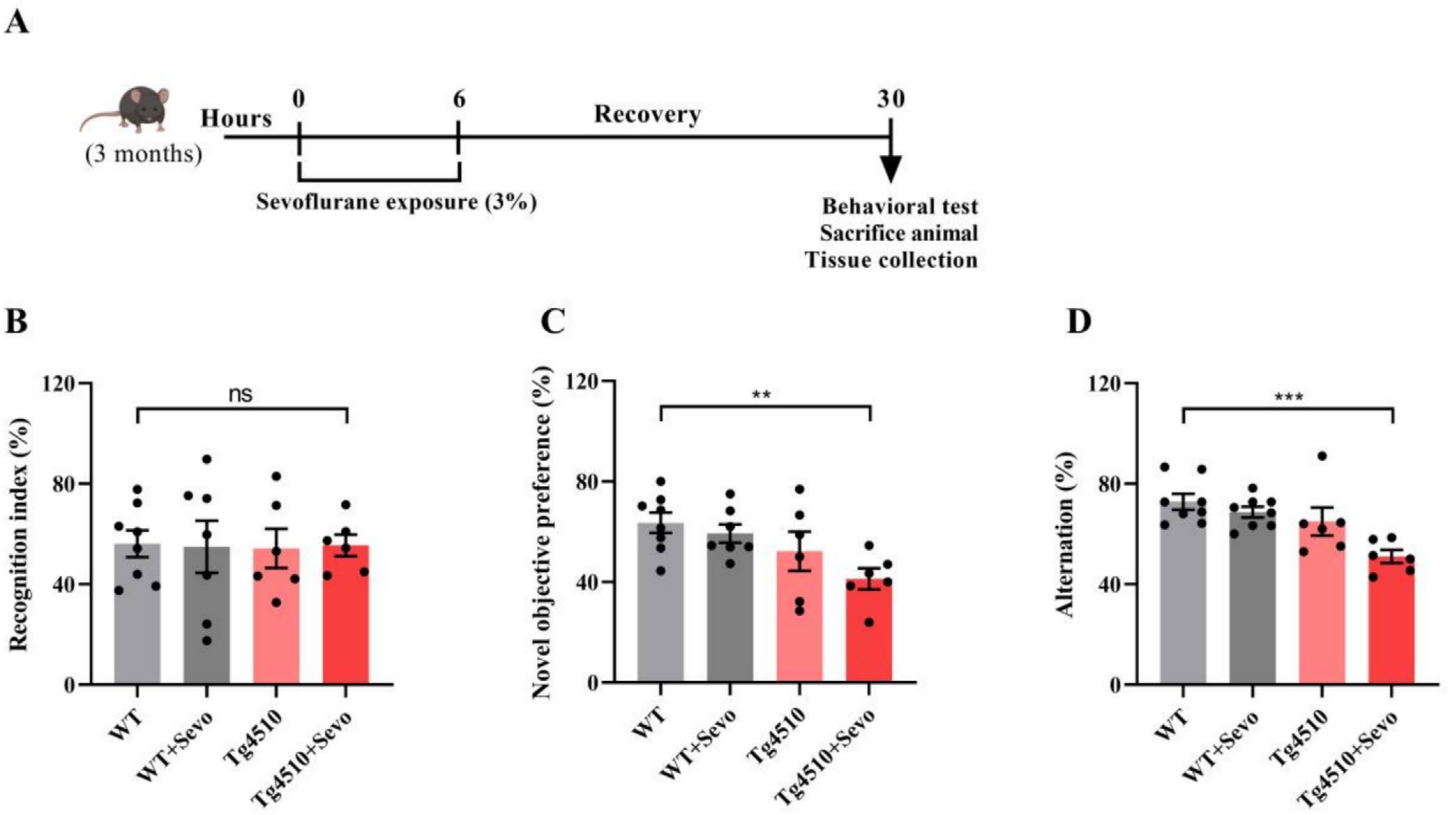
Sevoflurane exposure aggravates cognitive impairment of Tg4510 mice. (A) Sevoflurane exposure diagram and experimental approach. (B) The recognition index in the training period of the novel object recognition test (*n* = 6–8). (C) The percentage of novel object preference in the detection period of the novel object recognition test (*n* = 6–8). (D) Spontaneous alternation in the Y‐maze task of Tg4510 mice (*n* = 6–8). Data are presented as mean ± SEM; **p < 0.01 and ***p < 0.001.

The mice were divided into four groups: WT, WT + Sevo, Tg4510, and Tg4510 + Sevo. The sevoflurane groups were exposed to 3% sevoflurane and 60% O_2_ for 6 h, while the control groups were exposed to 60% O_2_ for the same period. As depicted in previous study [30], the concentrations of sevoflurane and O_2_ were continuously monitored using an anesthetic analyzer (GE Healthcare, Munich, Germany). A detailed exposure strategy was shown in Figure 1A.

### 2.3 Novel Object Recognition Test

Novel object recognition test was conducted to evaluate the mouse's memory ability toward a novel object. During the training period, the experimental animal was permitted to explore the two identical objects freely for 10 min. After an interval of 60 min, one of the objects was replaced with a novel object in its place; mice were then allowed to explore the familiar and novel object for 5 min, and the interactions of the mouse with the objects were video recorded. An interaction was defined by the nose of the mouse pointing to the object within a distance of 1.5 cm. Time spent exploring each object was recorded using the EthoVision XT 16.0 software (Noldus, the Netherlands).

### 2.4 Y‐Maze Test

Y‐maze test was adapted to assess the short‐term spatial working memory. The device consisted of three identical arms (44 × 10 × 15 cm) orientated at 120° angles from each other. According to the published protocols, the mouse was placed in the center of the apparatus and permitted to explore the arms for 10 min with no training. An alternation was operationally defined as the consecutive entry into all three arms. Subsequently, the number of arm entries and alternations was documented to evaluate the percentage of the alternation behavior. The mouse exhibiting strong working memory capabilities demonstrated a propensity to enter the arms of a maze that had not been recently visited. A higher percentage of alternation was indicative of a stronger working memory, as it suggested that the mouse was able to recall which arms it had previously visited.

### 2.5 Immunofluorescence

The brain sections from WT mice and Tg4510 mice with or without sevoflurane exposure are used to measure the change of tau pathology, neuroinflammation, and autophagy. Detailed information is mentioned in Appendix S1.

### 2.6 Proteome and Bioinformatics Analysis

A proteomics study was performed as previously reported [31]. Detailed information is provided in Appendix S1, including protein preparation, LC–MS/MS, and bioinformatic analysis.

### 2.7 Western Blotting

The tissues were lysed with RIPA lysis buffer containing protease and phosphatase inhibitors, followed by centrifugation and protein quantification. All primary antibodies (Table 1) were used to assess tau pathology (Tau 5, AT8, AT180, AT270, Tau pS396, and Tau pS199), synaptic defects (PSD95, synapsin‐1, SNAP25, and syn), neuroinflammatory response (GFAP and Iba1), and autophagy‐related signaling (Sort1, Vps28, Atg3, p62, p‐AKT, AKT, p‐mTOR, mTOR, and p‐AMPK). Detailed information is mentioned in Appendix S1.

**TABLE 1:** The primary antibodies used in this study.

Antibody	Cat.	Type	Application	Source
AT8	MN1020	Mouse	WB/IF	Thermo
AT180	MN1040	Mouse	WB/IF	Thermo
AT270	MN1050	Mouse	WB	Thermo
Tau 5	ab80579	Mouse	WB	Abcam
PSD95	2507S	Rabbit	WB	Cell Signaling Technology
Synapsin‐1	ab254349	Rabbit	WB	Abcam
SNAP25	ab109105	Rabbit	WB	Abcam
Syn	ab32127	Rabbit	WB	Abcam
GFAP	MAB360	Mouse	WB/IF	Merck
Iba1	019–19,741	Rabbit	WB/IF	Wako
Sort1	A7926	Rabbit	WB	ABclonal
Vps28	A9104	Rabbit	WB	ABclonal
Atg3	A5809	Rabbit	WB	ABclonal
p62	ab109012	Rabbit	WB/IF	Rabbit
p‐AKT	4060S	Rabbit	WB	Cell Signaling Technology
AKT	4691S	Rabbit	WB	Cell Signaling Technology
p‐mTOR	2971S	Rabbit	WB	Cell Signaling Technology
mTOR	2972S	Rabbit	WB	Cell Signaling Technology
p‐AMPK	2535S	Rabbit	WB	Cell Signaling Technology
β‐Actin	sc‐47,778	Mouse	WB	Santa Cruz

### 2.8 Statistical Analysis

Data were expressed as the mean ± SEM and analyzed using GraphPad Prism 8.0 statistical software (GraphPad Software Inc., La Jolla, CA, USA). Student's *t*‐test was used to assess the statistical significance between two groups. The Shapiro–Wilk normality test was used to assess the normal distribution of the data. When the data were normally distributed, one‐way analysis of variance (ANOVA) followed by Dunnett's multiple comparison test was conducted to evaluate the level of significance among multiple groups. Statistical significance was set as *p < 0.05, **p < 0.01, and ***p < 0.001.

## 3 Results

### 3.1 Sevoflurane Exposure Exacerbates Short‐Term Cognitive Impairment in Tg4510 Mice

As reported in previous studies, we revealed a significant increase in the onset of cognitive impairment associated with sevoflurane exposure in the context of Aβ pathology [8]. Considering the crucial role of tau pathology in the pathogenesis of AD, we further assessed the effect of sevoflurane exposure on tau pathology‐related cognitive impairment in Tg4510 mice. After being exposed to 3% sevoflurane, the 3‐month‐old Tg4510 mice were allowed to recover for 24 h, followed by performing cognitive‐related behavioral experiments (Figure 1A). As indicated by the results of the novel object recognition test, there was no significant difference in the preference for the novel object between the Tg4510 mice and the WT mice, while sevoflurane exposure largely decreased the cognitive ability for the novel object of Tg4510 mice compared to WT mice (Figure 1B,C). Similarly, further reduction in the percentage of alternation in the Y‐maze test was also observed in Tg4510 mice exposed to sevoflurane compared to WT mice (Figure 1D). Since novel object recognition and the Y‐maze are usually considered to evaluate short‐term memory, these findings in the present study indicate that sevoflurane exposure exacerbates short‐term cognitive impairment in the early stage of Tg4510 mice.

### 3.2 Sevoflurane Exposure Significantly Increases Pathological Changes of Tau in the Brain of 3‐Month‐Old Tg4510 Mice

**FIGURE 2:**
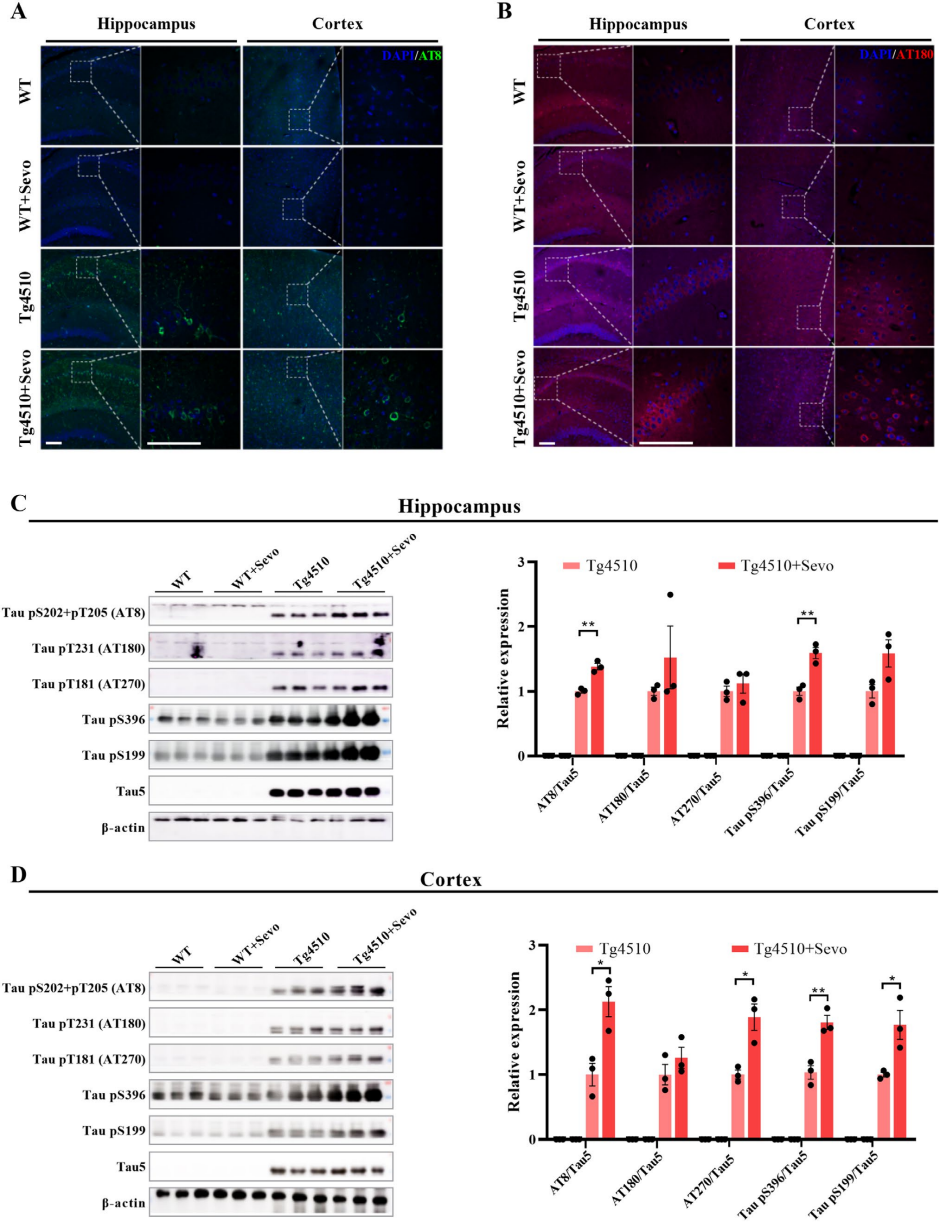
Sevoflurane exposure exacerbates tau phosphorylation of Tg4510 mice. (A, B) Images of the hippocampal and cortical tissue slices stained for AT8 antibody (green) and AT180 (red) from WT, WT + Sevo, Tg4510, and Tg4510 + Sevo mice. Scale bar: 100 μm. (C) Representative images and quantitative analyses of total tau and tau phosphorylation associated proteins in the hippocampus, including AT8, AT180, AT270, Tau pS396, Tau pS199, and Tau 5 (*n* = 3). (D) Representative images and quantitative analyses of total tau and tau phosphorylation associated proteins in the cortex (*n* = 3). Data are presented as mean ± SEM; *p < 0.05 and **p < 0.01.

Tg4510 mice express a repressible human tau protein that contains the P301L mutation associated with familial frontotemporal dementia. In general, spatial memory impairment in this model of mice appears between 2.5 and 4 months of age [32, 33], while the progressive tau pathology begins at 2.5 months of age and develops neurofibrillary tangles (NFTs), neuronal deficits, and brain atrophy by 6 months of age [34, 35]. Thus, we next assess the effect of sevoflurane exposure on tau pathology in the early stage of Tg4510 mice. The results of immunofluorescence showed that sevoflurane exposure increased the expression of AT8 and AT180 in the hippocampus and cortex of Tg4510 mice compared to the nonexposed Tg4510 group (Figure 2A,B). Meanwhile, these findings were further confirmed as indicated by the elevated expression of AT8 and Tau pS396 in hippocampal tissues and AT8, AT270, Tau pS396, and Tau pS199 in cortical tissues (Figure 2C,D). Overall, our data suggest that sevoflurane exposure exhibits a strong promotion in the progression of tau pathology.

### 3.3 The Significantly Synaptic Defects Were Observed in Cortex and Hippocampal Brain Regions of 3‐Month‐Old rTg4510 Mice Exposed to Sevoflurane

**FIGURE 3:**
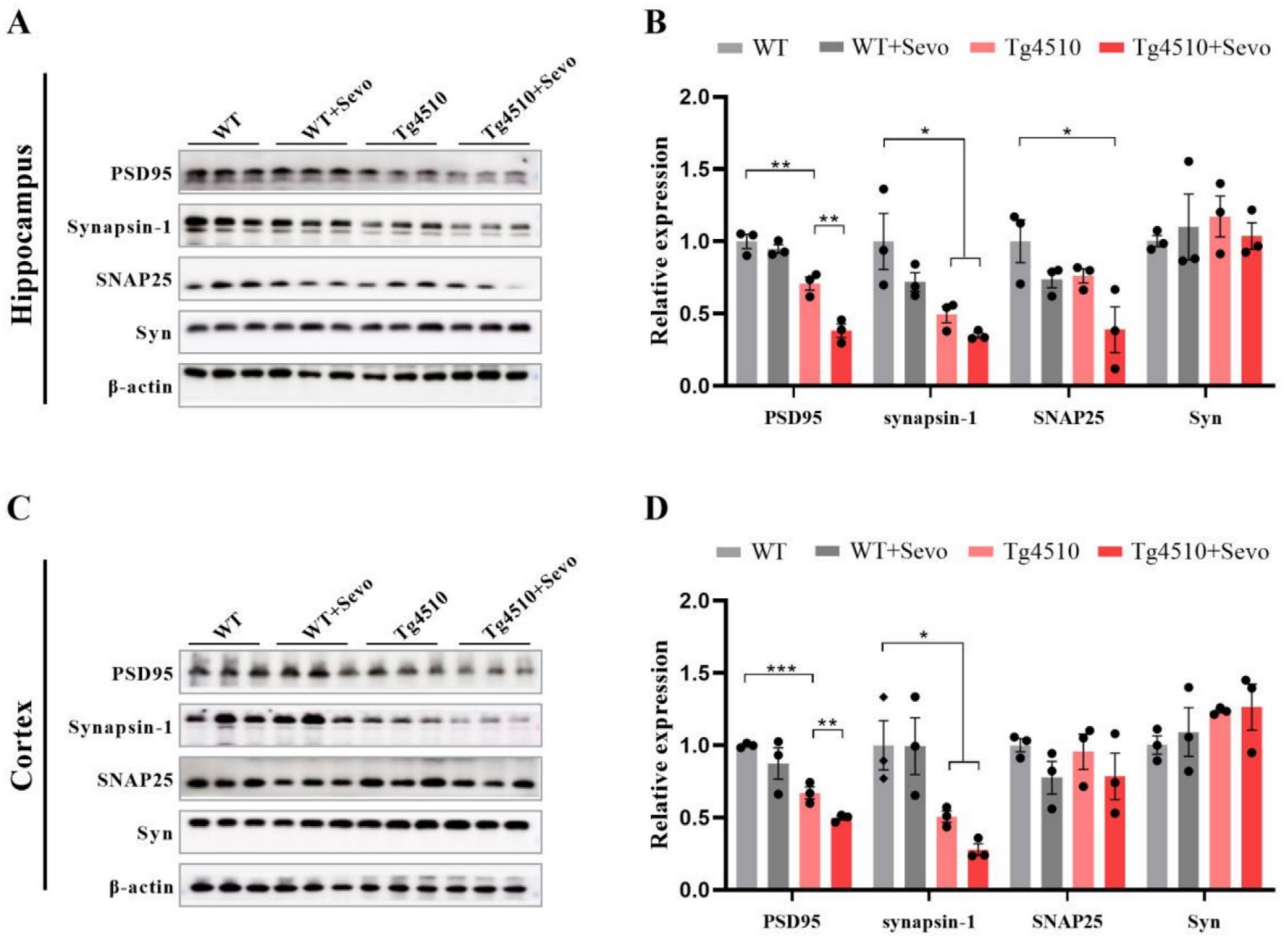
Sevoflurane exposure decreases the expression of synaptic‐associated proteins of Tg4510 mice. (A–D) Representative images and quantitative analyses of synaptic‐associated proteins (including PSD95, synapsin‐1, SNAP25, and syn) in the hippocampus and cortex from WT, WT + Sevo, Tg4510, and Tg4510 + Sevo mice (*n* = 3). Data are presented as mean ± SEM; *p < 0.05, **p < 0.01, and ***p < 0.001.

Given the crucial role of synaptic degeneration in cognitive function [36], we measured the expression of synaptic proteins such as PSD95, synapsin‐1, SNAP25, and synaptophysin (Syn) in the hippocampus and cortex of the four groups of mice. The results showed that the expression of PSD95 and synapsin‐1 was significantly decreased in the hippocampal and cortical tissues of Tg4510 mice exposed to sevoflurane (Figure 3). Additionally, we also found that the expression of SNAP25 was further reduced in the hippocampus of sevoflurane‐exposed Tg4510 mice (Figure 3A,B). Thus, the evidence of the above data supports the notion of sevoflurane‐exacerbating synaptic defects in the early stage of Tg4510 mice.

### 3.4 Sevoflurane Predominantly Upregulates Neuroinflammatory Response in the Brian of Tg4510 Mice

**FIGURE 4:**
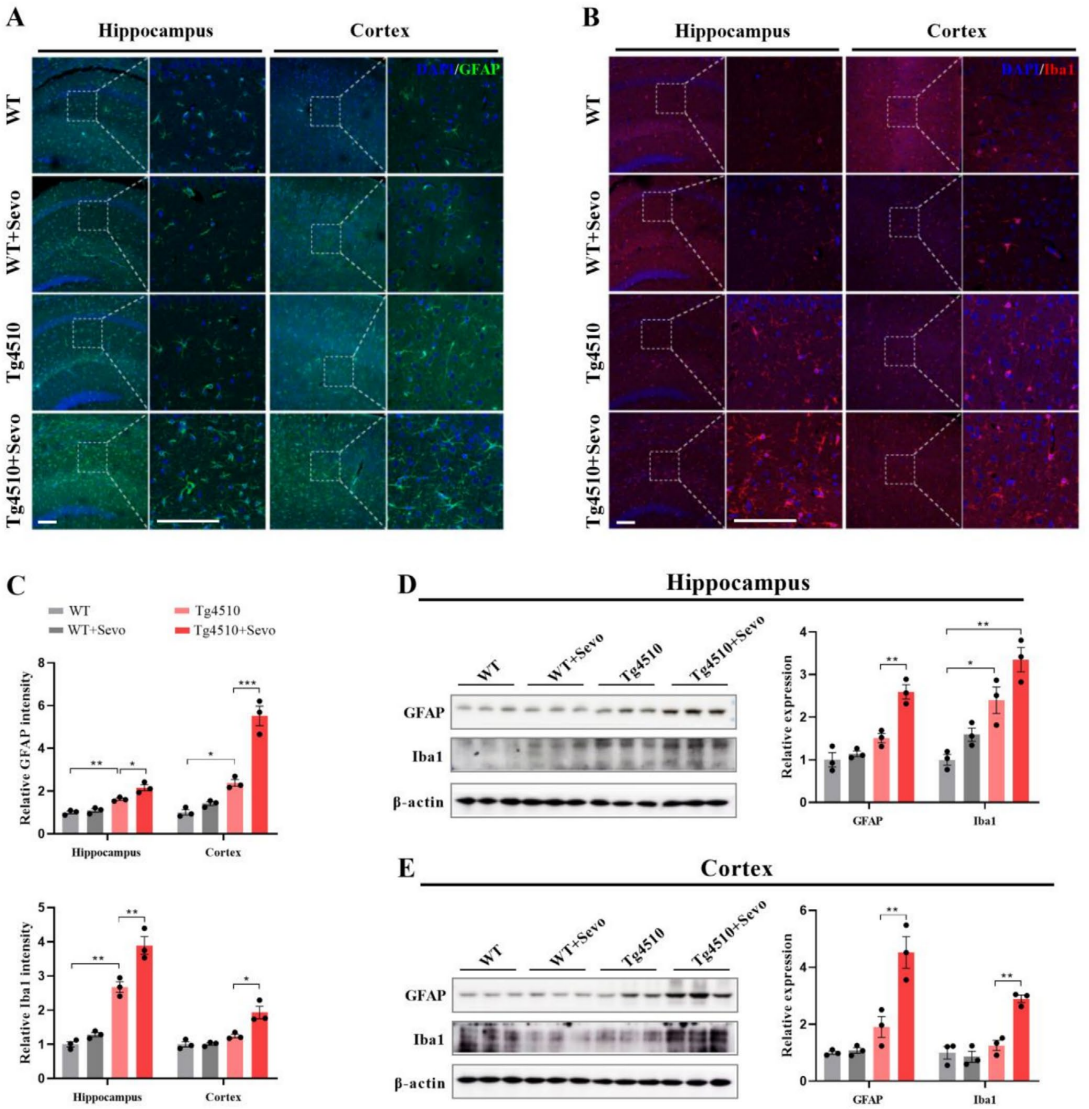
Sevoflurane exposure upregulates the neuroinflammatory response of Tg4510 mice. (A–C) Representative immunofluorescence images and quantitative graphs showing the expression of GFAP and Iba1 in the hippocampus and cortex. Scale bar: 100 μm (*n* = 3). (D, E) Western blot analysis of GFAP and Iba1 in the hippocampus and cortex from WT, WT + Sevo, Tg4510, and Tg4510 + Sevo mice (*n* = 3). Data are presented as mean ± SEM; *p < 0.05, **p < 0.01, and ***p < 0.001.

Neuronal gliosis is one of the most important pro‐inflammatory mechanisms in AD pathology, which is closely associated with the activation of glial cells (astrocytes and microglia) [37]. Although the causal relationship between gliosis and the progression of tau pathology remains unclarified, there is no doubt that the activation of reactive glial cells is an important cause of tau pathology‐driven cognitive impairment. In this study, a significant increase in glial cellular markers (GFAP/Iba1) was detected in the hippocampal and cortical tissues of Tg4510 mice with sevoflurane exposure (Figure 4A–C). Meanwhile, immunoblotting analysis also revealed an obvious increase in the expression of GFAP and Iba1 in the hippocampus of sevoflurane‐exposed Tg4510 mice (Figure 4D,E). Thus, these findings suggest that sevoflurane exposure promotes the neuroinflammatory response by activating neuronal gliosis, which may contribute to the progression of cognitive impairment of Tg4510 mice.

### 3.5 Proteomics Analysis Reveals Significant Dysregulation of Autophagy and Immune Inflammation‐Related Processes in the Hippocampus of Tg4510 Mice Exposed to Sevoflurane

**FIGURE 5:**
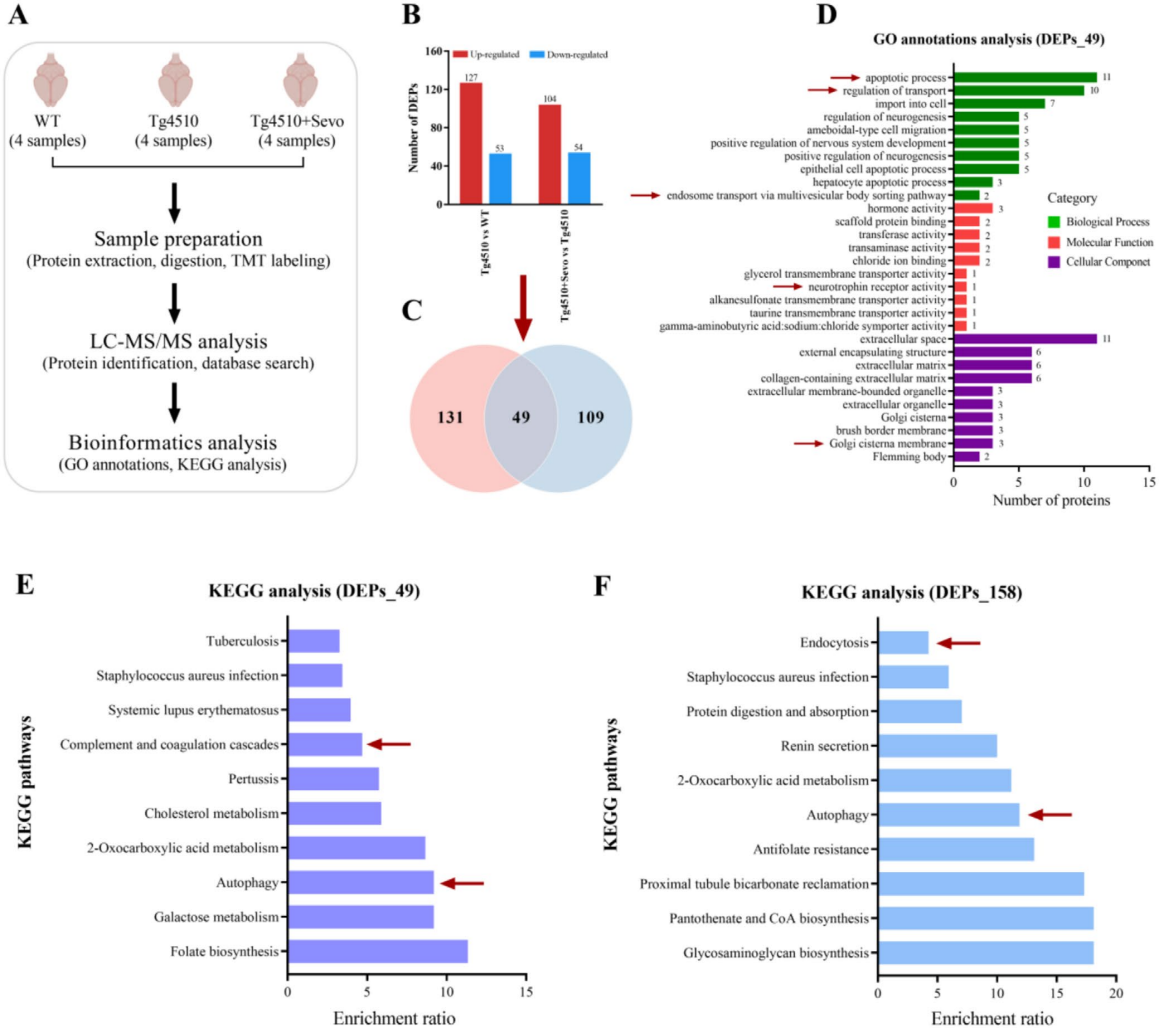
Sevoflurane exposure induces significant dysregulation of autophagy and immune inflammation‐related processes in the hippocampus of Tg4510 mice. (A) Schematic diagram of proteomics analysis. (B) Quantitative graphs showing the differentially expressed proteins (DEPs, fold change > 1.2) in the hippocampus of Tg4510 versus WT and Tg4510 + Sevo versus Tg4510 mice. (C) The Venn analysis of DEPs among three groups. (D) The GO analysis of shared DEPs based on biological process, molecular function, and cellular component. (E) The KEGG analysis of shared DEPs. (F) The KEGG analysis of DEPs in the hippocampus of Tg4510 mice with or without sevoflurane exposure.

Next, to explore the underlying mechanisms of sevoflurane‐induced cognitive deterioration associated with tau pathology, TMT‐labeled proteomics analysis was performed in the hippocampus of three groups of mice (Figure 5A). A total of 7273 proteins were identified across the three experimental groups (Table S1), and subsequently screened for the differentially expressed proteins (DEPs) according to the fold change ≥ 1.2. As shown in Figure 5B, we found that a total of 180 proteins (127 upregulated, 53 downregulated) were significantly differentially expressed in the hippocampus of Tg4510 mice compared to the WT mice, whereas 158 DEPs (104 upregulated, 54 downregulated) were detected in the hippocampus of sevoflurane‐exposed Tg4510 mice compared to the nonexposed Tg4510 mice. Of these, a total of 49 DEPs were shared among three groups (Figure 5C), which might be the molecular basis for sevoflurane‐exacerbating tau pathology‐related cognitive decline. Therefore, we performed GO annotations analysis and KEGG analysis for the 49 DEPs. GO analysis indicated that the 49 DEPs were mainly enriched in apoptotic process, regulation of transport, endosome transport via multivesicular body sorting pathway, neurotrophin receptor activity, and Golgi cisterna membrane (Figure 5D). Specifically, the DEPs involved in endosome transport via multivesicular body sorting pathway, neurotrophin receptor activity, and Golgi cisterna membrane mainly included Sort1, Vps28, Chsy3, and Golim4, which were linked with the initiation of autophagy and all downregulated in the hippocampus of Tg4510 mice exposed to sevoflurane, suggesting the involvement of autophagy in sevoflurane‐induced cognitive impairment. Meanwhile, KEGG analysis revealed that the 49 DEPs were mainly involved in autophagy and complement and coagulation cascades (Figure 5E). The DEPs related to autophagy mainly included Atg3 and Ulk2, which were significantly downregulated in sevoflurane‐exposed Tg4510 mice compared to the nonexposed Tg4510 mice. Moreover, the autophagy and endocytosis pathways were enriched in KEGG analysis for the 158 DEPs associated with sevoflurane exposure in Tg4510 mice (Figure 5F). These findings strongly suggest the crucial role of autophagy in sevoflurane‐induced cognitive impairment of Tg4510 mice. Besides, the pathway of complement and coagulation cascades associated with immune inflammation was also significantly enriched in KEGG analysis for the above 49 DEPs, which was consistent with the results of the activation of glial cells caused by sevoflurane exposure. Thus, this evidence indicates that downregulated autophagy and elevated neuroinflammatory response may be the key processes in which sevoflurane aggravates cognitive degradation in Tg4510 mice.

### 3.6 AKT/mTOR‐Mediated Autophagy Signaling Is Inhibited in Sevoflurane‐Exposed Tg4510 Mice

**FIGURE 6:**
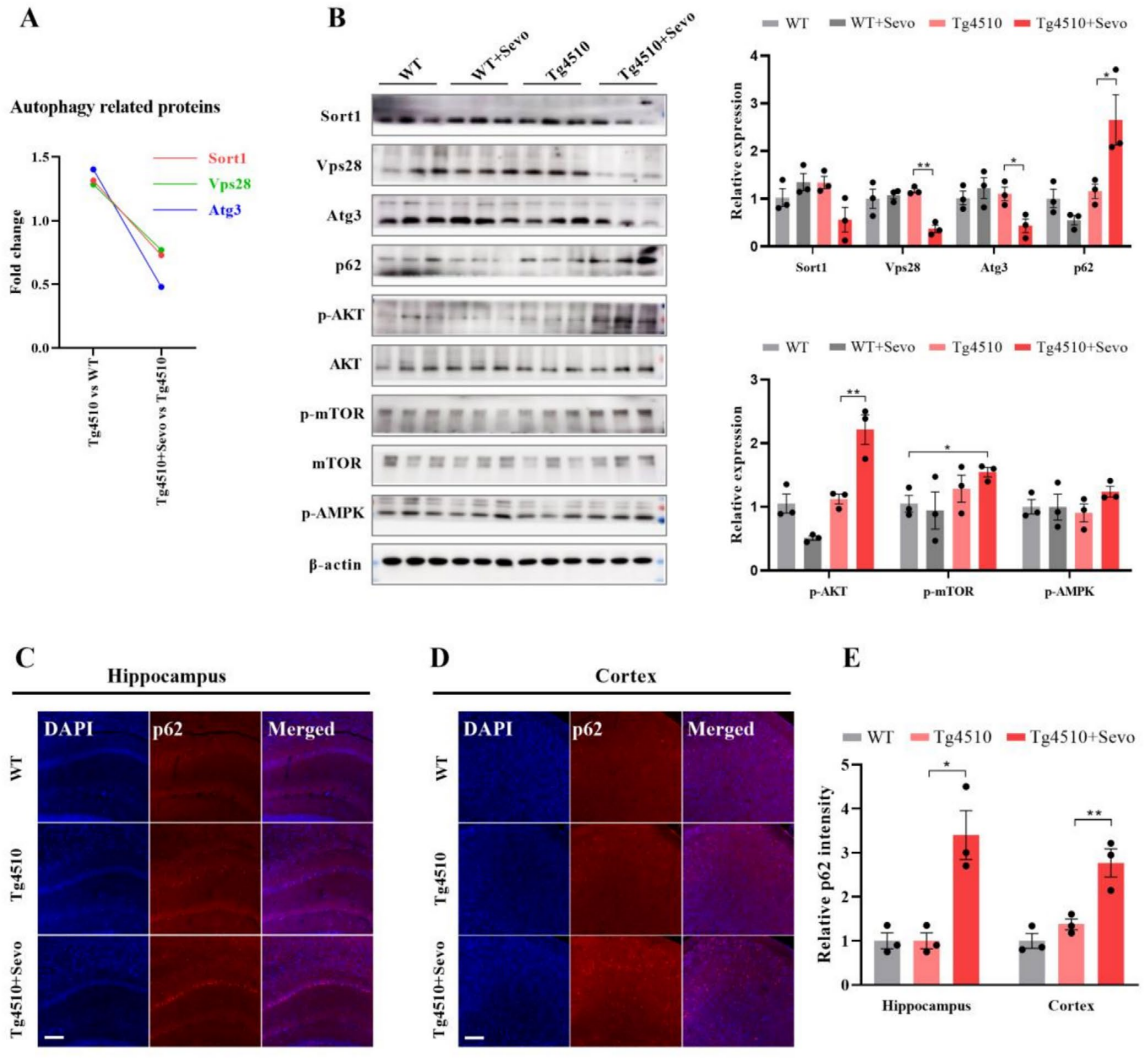
Sevoflurane exposure significantly suppresses AKT/mTOR‐mediated autophagy signaling in Tg4510 mice. (A) The change of autophagy‐related proteins between Tg4510 versus WT and Tg4510 + Sevo versus Tg4510 in the proteomics experiment. (B) Western blot analysis of proteins associated with autophagy signaling (*n* = 3). (C–E) Representative immunofluorescence images and quantitative graphs showing the expression of p62 in the hippocampus and cortex. Scale bar: 100 μm (*n* = 3). Data are presented as mean ± SEM; *p < 0.05 and **p < 0.01.

Autophagy is an important biological process involved in the clearance of abnormally aggregated proteins and dysfunctional organelles, and markedly impaired in AD [38]. In this study, we firstly validated the expression of autophagy‐related proteins identified by the proteomics. The results showed that there was no significant difference in the expression of Sort1, Vps28, and Atg3 between Tg4510 mice and WT mice, while the expression of the above key proteins was decreased in the Tg4510 mice exposed to sevoflurane (Figure 6A,B). Additionally, the assessment of the hallmark proteins of autophagy demonstrated an inhibiting effect of sevoflurane exposure on autophagy, as indicated by the increased expression of p62 in the brain of Tg4510 mice exposed to sevoflurane (Figure 6B–E). Thus, these findings further supported the notion that downregulation of autophagy may play a pivotal role in sevoflurane‐exacerbating cognitive degradation.

Importantly, the occurrence and development of autophagy are regulated by various signaling pathways such as the AKT/mTOR pathway. Previous studies demonstrated that inhibition of the AKT/mTOR signaling pathway could activate autophagy, thereby inducing protective effects [39]. Our data revealed that the levels of p‐AKT and p‐mTOR were significantly elevated in the hippocampus of Tg4510 mice exposed to sevoflurane, while there was no obvious difference in the expression of p‐AMPK between the two groups (Figure 6B). Collectively, these findings indicate that sevoflurane exposure inhibits the autophagy of Tg4510 mice mainly through activating AKT/mTOR rather than the AMPK/mTOR pathway.

## 4 Discussion

In this study, our data revealed a strong promotion for sevoflurane‐exacerbating cognitive degradation in the early stage of Tg4510 mice. In addition, the increased tau phosphorylation, synaptic defects, and neuroinflammation further emphasized the adverse effects of sevoflurane on P301L tau transgenic mice, which contributed to the progression of AD‐related cognitive impairment. Mechanistically, we found that sevoflurane exposure could inhibit the autophagy process by activating the AKT/mTOR pathway. Considering the crucial role of autophagy in AD, we speculated that targeting AKT/mTOR‐mediated autophagy signaling may offer novel therapeutic avenues for sevoflurane‐induced cognitive impairment associated with tau pathology.

Although there is still controversy about whether sevoflurane exposure promotes cognitive impairment in clinic settings, a large number of animal studies show that sevoflurane plays an important role in exacerbating cognitive impairment. For instance, sevoflurane was found to induce cognitive dysfunction in young and postoperative cognitive dysfunction (POCD) mice [40, 41]. Besides, emerging evidence also demonstrated the strong contribution of sevoflurane to the onset and progression of cognitive impairment in the context of AD‐related risk factors [8, 30, 42]. Our previous studies showed that sevoflurane exposure could accelerate the onset of cognitive impairment and aggravate the neuroinflammatory response in Aβ model mice [8, 9]. In this study, we found that sevoflurane exposure exacerbated the appearance of cognitive impairment in 3‐month‐old Tg4510 mice. The above evidence further supports the notion that sevoflurane has a powerful promoting effect in inducing the emergence of cognitive impairment under the AD pathology conditions. However, the question of whether the effect of sevoflurane in accelerating cognitive impairment is irreversible still deserves in‐depth study. But there is no doubt that sevoflurane exposure exacerbates the neurotoxicity associated with tau pathology.

Tau, a microtubule‐associated protein, is mainly responsible for stabilizing microtubules and promoting axonal transport. Normally, tau protein is soluble and natively unfolded, while it becomes insoluble aggregates called neurofibrillary tangles when hyperphosphorylated [43]. Tau hyperphosphorylation is reported to be involved in the regulation of multiple other neurodegenerative diseases, including frontotemporal dementia with parkinsonism‐17 (FTDP‐17), Pick disease (PiD), argyrophilic grain disease (AGD), and corticobasal degeneration (CBD), which are collectively known as tauopathies [44]. In this study, we found that sevoflurane exposure significantly increased the phosphorylation at a variety of sites of tau protein in Tg4510 mice. In line with the results of this study, recent work revealed that sevoflurane could induce sequential tau phosphorylation in the developing brain [6]. Thus, it is reasonable to speculate that sevoflurane exposure may have similar neurotoxic effects in other tauopathies, not just limited to AD.

In addition, the glial cells mediated neuroinflammation associated with the activation of astrocytes and microglia is considered the initiator of toxic tau protein and Aβ deposition in AD [45]. Meanwhile, increasing evidence demonstrates that the enhancement of pro‐inflammatory signaling is involved in the regulation of synapse loss [46, 47], suggesting that neuroinflammation plays a pivotal role in synaptic defects and cognitive impairment. Herein, our study revealed that sevoflurane exposure could induce a neuroinflammatory response in 3‐month‐old Tg4510 mice, which may be one of the causes of sevoflurane‐exacerbating cognitive impairment. Additionally, sevoflurane exposure also demonstrated a significant increase in synaptic defects in this study. The above evidence further highlighted the strong association between sevoflurane exposure and neuroinflammation. However, the deterioration of tau pathology further exacerbates AD‐related neuroinflammation with the progression of disease, thereby forming a vicious cycle between tau pathology and neuroinflammation [48]. Therefore, the exact mechanism of sevoflurane in this vicious cycle is worth further investigation.

Mechanistically, our study found that sevoflurane exposure significantly inhibited the autophagy process in Tg4510 mice, as indicated by the decreased expression of Sort1, Vps28, and Atg3 in proteomics analysis. Interestingly, Sort1 is originally known to be associated with lipid metabolism [49]. A recent study showed that the elevated expression of Sort1 contributed to enhancing autophagic flux in both transformed and primary hepatic cells [50]. Additionally, numerous studies have also confirmed the critical role of autophagy‐related proteins Vps28 and Atg3 in the initiation of autophagy [51, 52]. These pieces of evidence further highlight the important relationship between autophagy inhibition and sevoflurane exposure. Given the key role of autophagy in the clearance of toxic proteins such as phosphorylated tau and Aβ, we speculated that sevoflurane‐induced cognitive impairment associated with tau pathology might be related to the altered autophagy. Besides, the activation of autophagy is regulated by a variety of upstream signals. Of these, mTOR, a serine/threonine kinase, is a master regulator of cellular autophagy [53]. Inhibition of mTOR signaling is one of the most potent inducers of autophagic activity [54]. A large number of physiological signals affect the activation or inhibition of mTOR, such as AMPK, AKT, and growth factor signaling (GFS) [55, 56]. In this study, we found that sevoflurane exposure activated the AKT/mTOR signaling rather than the AMPK/mTOR pathway, suggesting that AKT/mTOR‐mediated autophagy inhibition may be a pivotal process in sevoflurane‐induced cognitive impairment.

## 5 Conclusions

In conclusion, our study demonstrates that sevoflurane exposure significantly exacerbated the progression of cognitive impairment in the Tg4510 mice. Moreover, the deterioration of pathological manifestations, including tau phosphorylation, synaptic defects, and neuroinflammatory response, is observed in sevoflurane‐exposed Tg4510 mice. Further, we found that sevoflurane‐induced cognitive impairment was linked with the inhibition of the AKT/mTOR‐mediated autophagy pathway, suggesting that interventions for this pathway might be a potential target for developing therapies to combat sevoflurane‐induced cognitive decline associated with tau pathology.

## Author Contributions

Kaiwu He designed and performed the experiments and wrote the manuscript. Zhijing Zhang and Youzhi Li analyzed the data. Wei Xiong, Yanmei Xing, Wenli Gao, Wei Kong, and Lixin Chen helped with the manuscript writing, experimental tools, and supported the study. Zhongliang Dai and Xifei Yang helped in the design of the experiments and the editing of the manuscript. The corresponding authors reviewed and approved the manuscript and held all the responsibilities related to this manuscript. All authors reviewed and approved the manuscript.

## Funding

This work was supported by the National Natural Science Foundation of China (82471629, 82301748), Natural Science Foundation of Guangdong Province (2022A1515012129), Shenzhen Municipal Science and Technology Foundation (JCYJ20220530152615035, JCYJ20240813103900001, JCYJ20240813104600001), Sanming Project of Medicine in Shenzhen (SZSM202211010), Shenzhen Key Medical Discipline Construction Fund (SZXK069), and Shenzhen Medical Research Fund (C2501030).

## Ethics Statement

All experimental procedures were performed according to the protocols approved by the Animal Care and Use Committee of Shenzhen People's Hospital (approval number: AUP‐220516‐DZL‐0230‐01).

## Conflicts of Interest

The authors declare no conflicts of interest.

## Supporting information


**Appendix S1:** cns70694‐sup‐0001‐AppendixS1.docx.

**Table S1:** cns70694‐sup‐0002‐TableS1.xlsx.

## Data Availability

The data that support the findings of this study are available from the corresponding author upon reasonable request.
