# Supplementary material for: Sevoflurane Exposure Exacerbates Memory Impairment and Pathological Manifestation by Inhibiting AKT/mTOR‐Mediated Autophagy in P301L Tau Transgenic Mice
Source: CNS Neurosci Ther. 2025 Dec 17;31(12):e70694. doi: 10.1002/cns.70694 (PMC12710523; doi:10.1002/cns.70694)
Supplement: Supplementary file 1 — Appendix S1: cns70694‐sup‐0001‐AppendixS1.docx. [file CNS-31-e70694-s002.docx]

**Proteomic analysis**

*Protein preparation, labeling and peptide fractionation*

Mice hippocampal tissue samples from WT mice and Tg4510 mice with or without sevoflurane exposure were lysed with 8 M urea, centrifuged at 4℃, 14000 g for 30 min, followed by collected into a new 1.5 ml centrifuge tube, and then collected the protein supernatant. Subsequently, 100 μg of protein was taken from each sample in the same group and pooled to obtain a system with a total of 100 μg protein per group. The three pooled protein samples were treated with 10 mM DL-dithiothreitol and 25 mM alkylated with 3-indoleacetic acid in turn. Each fully denatured proteins were then digested with 5 μg Trypsin/Lys-C Mix at 37℃ for 14 h. After digestion, the pooled samples were desalted by using peptide desalting spin columns, followed by drying and labeling with TMT 10plex reagents (90110, Thermo, USA).

TMT-labeled peptides were fractionated by using nanoflow DIONEX UltiMate 3000 RSLCnano System (Thermo Fisher Scientific, USA) coupled with a C18 column (300 Å, 5 μM). The samples were chromatographically separated by a gradient from 5% to 90% ACN at a flow rate of 0.3 μL/min. All peptide samples were separated into 60 fractions, which were combined into 15 fractions. Subsequently, these fractions were dried, re-dissolved and further analyzed by using LC-MS/MS.

*LC-MS/MS and database searching*

The redissolved peptide samples were analyzed using an Easy-nanoLC system coupled with the Orbitrap Exploris 480 mass spectrometer (ThermoFisher, USA). 1 μL of sample was injected for nanoLC-MS/MS analysis. Raw data was collected in data-dependent acquisition mode, with full scan mass spectra (m/z 350–1800) obtained at 70000 resolution in the Orbitrap detector after ion accumulation to a target value of 1×10^6^. A 20 ppm mass error tolerance was applied to both full scan and MS/MS spectra.

The raw files obtained from mass spectrometry were queried against a *Mus musculus* Uniprot database ^1^ (downloaded on July 20, 2023) using protein sequest within Proteome Discoverer (v2.6) software. Trypsin was designated as the protease with a maximum allowance of two missed cleavages. MS/MS spectra were analyzed with a precursor mass tolerance of 10 ppm and a fragment mass tolerance of 0.02 Da. Dynamic modifications of methionine oxidation and N-terminal acetylation were chosen, while carbamidomethyl cysteine was selected as a static modification. The thresholds for peptide and protein identifications, including PSM, protein, and site decoy fraction FDR, were established at 0.01. The quantitative data were presented in terms of protein abundance.

*Bioinformatic analysis*

Various proteomic analyses were conducted utilizing multiple methodologies. Proteins with fold change > 1.2 were defined as differentially expressed in the comparison group. To show the apparently altered biological processes in Tg4510 MICE with or without sevoflurane exposure. Venn analysis was employed to carry out a logistic analysis of the hippocampal proteome of two group mice. GO enrichment analysis based on biological process (BP), cellular component (CC) and molecular function (MF) and KEGG pathway analysis were conducted using the online Web-based Gene Set Analysis Toolkit (http://www.webgestalt.org) and further visualized using RStudio (v4.0.0).

**Immunofluorescence**

Paraffin-embedded tissue sections were deparaffinized using dimethyl benzene and subsequently rehydrated through a series of graded alcohol solutions. Antigen retrieval was performed using a citric acid buffer to unmask epitopes. Following three washes with PBS, the sections were blocked with a blocking buffer composed of 0.3% Triton X-100 and 3% bovine serum albumin in PBS for 60 min. The sections were then incubated overnight at 4℃ with primary antibodies, including mouse monoclonal anti-GFAP and rabbit polyclonal anti-Iba1. Sections were labeled with fluorescent secondary antibodies as follows: Alexa Fluor 488 goat anti-mouse IgG (H+L) and Alexa Fluor 568 goat anti-rabbit IgG (H+L). Nuclei were counterstained with DAPI (4,6-diamidino-2-phenylindole). Image acquisition was performed using LSM 980 (Zeiss, Germany) under a 100× objective and analyzed using ImageJ software.

**Western blotting**

Protein samples derived from animal tissues or cultured cells were extracted with RIPA lysis buffer supplemented with protease and phosphatase inhibitors (Thermo, USA). Subsequently, the proteins were separated via SDS-PAGE and transferred onto a PVDF membrane. The membrane was then blocked with 5% nonfat milk for 1 hour at room temperature, followed by sequential incubation with primary and secondary antibodies. Detailed information regarding the primary antibodies employed in this study is provided in Table 1. Protein level was detected using the PierceTM ECL Western Blotting Substrate kit (Thermo, USA) and quantified using ImageJ software.

**REFERENCES**

1. UniProt C. UniProt: the Universal Protein Knowledgebase in 2023. *Nucleic Acids Res.* 2023;51(D1):D523-D531.
